# Evolution of technology and disparities in antimicrobial stewardship: a narrative review

**DOI:** 10.1017/ash.2026.10404

**Published:** 2026-06-25

**Authors:** Christina Maguire, Ryan T. Clary, Oluchi Mbamalu, Jihye Kim, Kimberly B. Lee, Andrew Noda, Megan Backus, Barry Rittmann, Sangeeta Sastry

**Affiliations:** 1 Department of Pharmacy, Virginia Commonwealth University Health System, Richmond, USA; 2 Virginia Commonwealth University School of Medicine, Richmond, USA; 3 Division of Health Systems and Public Health, Faculty of Medicine and Health Sciences, Stellenbosch University, Capetown, South Africa; 4 Department of Internal Medicine, Division of Infectious Diseases, Virginia Commonwealth University Health System, USA

## Abstract

The number of resources devoted to antimicrobial stewardship programs (ASPs) has grown over the past thirty years as multidrug-resistant organism rates have increased. Technology plays a pivotal role in ASP expansion and allows for further reach, higher-yield initiatives, and improved patient safety and efficacy. This review highlights major technological innovations that have shaped ASPs over time and addresses the future of incorporating artificial intelligence into everyday ASP workflow. We discuss the current state of ASP technology in low- and middle-income countries with a focus on challenges and suggested solutions to incorporating artificial intelligence internationally. Bridging the global digital and technological divide through investments in resources (human capacity, infrastructure, and supporting environment) is vital to sustain progress against antimicrobial resistance.

## Graphical abstract

**Figure:**
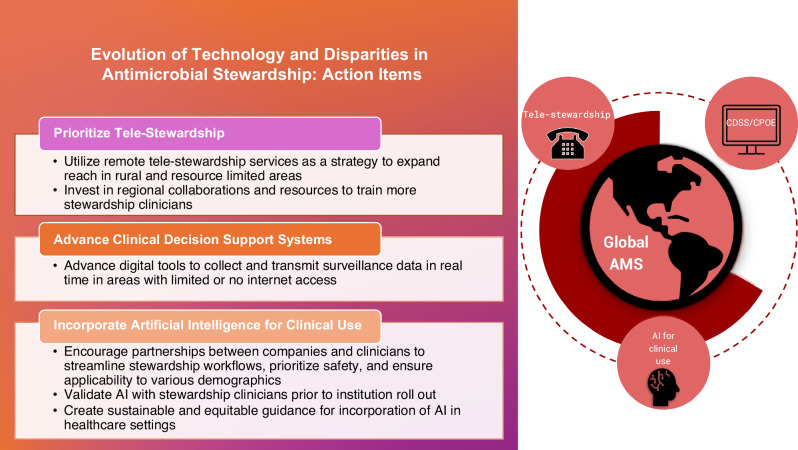

## Introduction

Recent advances in technology have enabled exponential growth of antimicrobial stewardship (AS) over the past thirty years. Newer guidelines encourage use of rapid diagnostics and antimicrobial use metrics with clinical decision support systems (CDSS).^1,2^ Despite these developments, access to technological resources varies internationally, underscoring the need for targeted national surveillance priorities and government support.^3^

Future advancements of AS lie with artificial intelligence (AI); data suggests AI improves antimicrobial resistance (AMR) predictions, optimizes antimicrobial selection, and streamlines AS workflows.^4^ While promising, AI implementation carries ethical, technical, and operational considerations.^5^ We describe technological advances in AS, highlighting the current state and need for equitable distribution of AS technology amongst low- and middle-income countries (LMIC).

## Evolution of technology in antimicrobial stewardship in high-income countries

### Background

In 2007, the first guideline for developing an AS institutional program underscored the need for instituting electronic medical records (EMRs), computerized provider order entry (CPOE), and CDSS.^1^ The Transatlantic Taskforce on Antimicrobial Resistance was founded to address AS best practices between the United States, European Union, United Kingdom, Norway, and Canada with projects like DebugIT paving the way to provide AMR prevention decision support.^6^

### Electronic medical records/computerized provider order entry

Prior to CPOE and EMRs, lack of real-time data stymied interventions. Stewards reviewed paper charts to evaluate therapeutic appropriateness and handwrote stewardship notes. With CPOE creation in the early 2000s–2010s, emphasis on AS was limited and required innovative approaches. Time-intensive CPOE customization alerted clinicians to restricted antimicrobial criteria and patients receiving antimicrobials and created stewardship screens with pertinent patient information.^7^ As targeted interventions were unavailable within CPOEs, third-party vendors identified organism-drug mismatch and de-escalation alerts.

### Clinical decision support systems

CDSS software layers clinical knowledge with patient-related information to present recommendations in real-time. First described in 1996, CDSS decreased antimicrobial cost, adverse events, and mortality through antimicrobial practice guidelines embedded into computer-assisted decision support.^8^ CDSS was associated with increased interventions, decreased time for interventions, and reduced hospital expenditures.^9^ Interventions included bug-drug mismatches, duration of therapy, and duplicate therapy. Financial restraints limited software integration into EMRs and customization to individual ASP needs. Stewardship clinicians spent two to three hours daily reviewing alerts and an additional one to two hours making interventions, leading to alert fatigue.^10^ Targeted digital interventions such as EMR alerts, dosing calculators, screening tools for *C. difficile* risk, and predictive models for treatment recommendations have become more common and are associated with reduced antimicrobial use, shorter hospital stays, and lower mortality.^11^

### Mobile applications

Resource development increased with widespread smartphone use. A systematic review evaluating the impact of smartphone or tablet applications on antimicrobial prescribing and guideline adherence highlighted that mobile device platforms were utilized more than desktop platforms. Mobile applications were associated with a significant increase in guideline adherence for common infections such as urinary tract infection and community-acquired pneumonia.^12^ However, only 34% of applications had medical professional involvement, leading the US Food and Drug Administration and European Commission to require regulatory oversight of any application intended to diagnose, treat, or cure a health issue in 2010.^13^

### Rapid diagnostic testing

Microbiological advances transformed the accuracy and speed of diagnosing infections. Mass spectrometry and single-plex polymerase chain reaction testing for quicker organism identification were first introduced many years ago, but implementation occurred only recently. Soon to follow were syndromic multiplex polymerase chain reaction for quick identification of infections; a meta-analysis noted that combining rapid diagnostic tests (RDT) with ASPs was associated with reduced mortality (OR 0.78; 95% CI 0.63–0.96) compared to RDT alone.^14^

### Tele-stewardship

Resource-limited hospitals often struggle to meet mandated stewardship requirements. Tele-ASP (remote, collaborative, and integrated models) is a strategy to achieve these goals.^15^ The optimal tele-ASP model is to establish a network with remote ID expertise and on-site pharmacist collaboration. For example, the Duke Antimicrobial Stewardship Outreach Network provides expert consultations, educational initiatives, data analysis, and feedback and integration to community hospitals without onsite ASPs.^16^ A step-by-step process for implementing remote infectious diseases consultation and AS in resource-limited settings highlights gaining key stakeholder support, creating institution-tailored procedures, assessing challenges to success, and establishing a sustainment phase as vital aspects.^17^

## Future of technology in ASP

As ASPs strive for greater precision, efficiency, and scalability, AI has emerged as a transformative tool. Particularly, machine learning (ML) models can optimize antimicrobial selection, predict AMR, personalize dosing regimens, and streamline workflows.

### Personal antibiograms/antimicrobial resistance prediction

An artificial neural network model was created to predict the likelihood of clinical success with empiric antimicrobial therapy for recurrent urinary tract infection.^18^ The model incorporated clinical and microbiologic data, prior antimicrobial exposures, personal antibiograms, and their associated susceptibility profiles to achieve a sensitivity of 87.8% and a specificity of 97.3% in predicting treatment outcomes. The study demonstrated that ML models can incorporate individualized patient factors to guide empiric antimicrobial selection and reduce inappropriate antimicrobial usage.

Another ML tool, called extreme gradient boosting (XGBoost), used decision trees to analyze emergency department data and predict urine culture susceptibility.^19^ The model achieved an area under the receiver operating curve (AUROC) of 74% after excluding negative cultures and positive cultures without susceptibility testing. Another XGBoost model used to predict AMR for Enterobacterales bloodstream infections found optimal empiric antimicrobial selection in 33% of cases compared to 26% at baseline, reduced antimicrobial consumption from 44% to 42%, and undertreatment from 30% to 25%.^20^ This model guided effective empiric decision-making solely by clinical metadata without requiring microbiologic species data.

The real-time prediction of drug resistance requires microbiologic, clinical, and demographic data. An ML algorithm that used 300,000 MALDI-TOF mass spectra and 750,000 resistance phenotypes to predict resistance without phenotypic susceptibility tests achieved an AUROC of 0.80 for MRSA and 0.74 for ceftriaxone-resistant *Escherichia coli* and *Klebsiella pneumoniae*.^21^ Similar models may be useful in areas that are unable to complete polymerase chain reaction genotypic testing.

CDSS can be combined with AI to reduce broad-spectrum antimicrobial prescribing. The Intelligent Stewardship Prompts to Improve Real-time Empiric Antibiotic Selection for Patients trials evaluated use of EMR prompts to prescribers when initiating antimicrobials in the first three days of hospitalization for management of various infections.^22^ The CDSS was created using recursive partitioning (supervised ML), evaluating patients for multidrug-resistant organism (MDRO) risk factors and providing recommendations for narrow-spectrum antimicrobials if patients were deemed low risk (<10%). The CDSS was associated with a significant decrease (17%–35%) in extended spectrum days of therapy (DOT) during the initial trial period; extended spectrum DOTs were reduced by up to 23% beyond the initial trial period, confirming clinician tendency to maintain initial therapy.

Based on available data, AI models show promise in assisting empiric antimicrobial decisions. Further progress and support from infectious disease experts is needed before widespread application to assess different patient populations and management of more nuanced antimicrobial decisions.

### Predictive analytics to streamline the workflow of ASPs

Given a shortage of stewardship clinicians and the rising use of tele-ASP, AI-driven recommendations for management of infections are of growing interest. A recent proof of concept study evaluated use of a GPT chatbot for blood culture result interpretations in an academic medical center.^23^ The authors provided external knowledge integration with both internal and published guideline recommendations and utilized chain of thought prompting. Chatbot recommendations were harmful or inadequate in 13% compared to 4% in the ASP group (*P* = .047). The chatbot performed well in RDT interpretation but underperformed in antimicrobial therapy and diagnostic recommendations. Given the myriad available ML approaches, an additional study investigated infection management accuracy of 14 large language models (LLMs).^24^ Large discrepancies between LLMs were identified; choice of antimicrobial was correct in 30%–72% of cases and dosage recommendations were correct in 27%–97% of cases. The authors highlighted the decreased accuracy of LLMs with increasingly complex cases and difficult-to-treat organisms. AI integrated into CDSS can improve AMR predictions and optimize antimicrobial choices, but current LLMs perform less effectively in complex clinical scenarios and produce substantial errors that could jeopardize patient safety.^4^

### Challenges and recommendations

AI implementation into HIC AS models faces multiple challenges (Table 1). As seen with the advent of CPOE and CDSS, ensuring data interoperability is paramount to successfully incorporating tools into the EMR and ensuring alert-based prompts are not extraneous or frequently overridden by clinicians. Lack of transparency of model logic, loss of accuracy in increasingly complex scenarios, and data sets based on specific populations limit current use of AI in AS. Lastly, high upfront costs for AI system development and validation in combination with major ethical and privacy concerns have slowed AI advances in healthcare.

**Table 1.** Summarizing challenges regarding the use and implementation of AI in high-income countries (HICs)

Challenge	Description
**EHR fragmentation and data interoperability**	Heterogeneous EHR ecosystems across institutions impede the standardized, large-scale data pipelines required for AI model training and implementation
**Alert fatigue from CDSS integration**	High volumes of AI- and rule-based alerts embedded in CPOE workflows are frequently overridden by clinicians, reducing the real-world effectiveness of AMS decision support
**Regulatory and liability concerns**	Evolving and inconsistent regulatory frameworks for AI-based clinical decision support create ambiguity about approval pathways, postmarket surveillance, and physician liability
**Model explainability and clinician trust**	Clinicians are reluctant to act on recommendations from opaque “black box” ML models, especially for high-stakes antibiotic decisions, limiting real-world adoption even with high model accuracy
**Implementation and maintenance costs**	The high upfront costs for AI system development coupled with need for validation, implementation, and ongoing maintenance can be difficult to justify for health systems
**Ethical and privacy considerations**	Use of large-scale patient data for AI training and inference raises concerns about data privacy, informed consent, and potential misuse of sensitive HIPAA information

Further incorporation of infectious disease experts into model building and transparency must take precedence; various patient populations must be tested (across demographics, rural vs urban regions, risk factors, severity of illness, etc.). When implementing models in healthcare systems, a stepwise approach should be considered; for example, trained stewardship clinicians should first use and validate software to ensure appropriateness before promoting institutionwide use. More risk averse integration, such as stewardship alerts, can be considered first.

## Technology in antimicrobial stewardship: current state in low- and middle-income countries

### Background

LMIC are disproportionately burdened by AMR. No international standards for AS existed until 2015, when the World Health Assembly created the Global Action Plan on Antimicrobial Resistance, outlining major tenets for action followed by the Pan American Health Organization stewardship guide and WHO toolkit for AS in LMIC.^3,25,26^ This toolkit suggested national steering committees develop targeted strategies as priorities may differ from country to country. Technology-related tenets mentioned include the need for national surveillance systems, increased lab capacity to guide optimal antimicrobial use, and improved availability of diagnostic tests.

The application of these technologies in LMIC remains limited, and operational enforcement of WHO national action plans is low.^27^ A 2023 study across seven Latin American and Caribbean countries found that while ASPs existed, many hospitals lacked formulary restriction, audit/feedback and leadership support.^28^ Similarly, in Southeast Asia, only 13.5% of survey respondents fulfilled all AS program requirements with a skew towards higher compliance in HIC countries; regarding technology, 49% had IT capabilities to analyze data, 64.7% had EHRs, and 56% had CPOES.^29^

Although the potential benefits such as improved surveillance, data-driven decision support, and optimized prescribing practices are clear for various tools, particularly AI, the implementation of such systems in resource-limited settings faces significant challenges, including human and infrastructural resource limitations.^30^ Additionally, the absence of high-quality datasets, critical for training AI models, further complicates integration of AI in ASPs.^30^ In many countries, contextual issues such as unreliable power supply and poor internet connectivity limit the transformative role that AI could play in AS.

### Challenges to implementation: technology and data limitations

Healthcare facilities in low-income countries generally lack access to EMRs, with many institutions relying heavily on paper-based systems; for example, in the Eastern Mediterranean region digital health action plan, low internet and smartphone access in low-income countries (LIC) was cited as a major barrier to investment in digital health resources.^31^ This complicates collection, analysis, and sharing of health data, hindering antibiotic prescription and resistance pattern monitoring. Additionally, diagnostic services are limited or non-existent, with digital diagnostic services even more so. Use of RDT is limited in LIC as it can be challenging to overcome the upfront costs as well as ensure the availability of personnel to intervene in a timely manner.^32^ Without diagnostic data, it is challenging to monitor resistance profiles, implement real-time feedback mechanisms, or adjust prescribing practices. In addition, out-of-pocket payment for care in such cases severely limits RDT use.^33^ Despite these challenges, the WHO highlights the need for “affordable, sensitive, specific and rapid diagnostic tests” to guide need and choice of antibiotics.^3^

In middle-income countries, the picture is more mixed. While there is a shift toward mobile health platforms and other digital tools, challenges such as fragmented health infrastructure, insufficient laboratory capacity, limitations in digital literacy, poor governance, and a shortage of trained personnel persist.^31^ The scalability of digital AS solutions remains constrained by these issues. The costs associated with their deployment, maintenance, and monitoring may exceed available resources budgeted by local institutions, limiting their long-term sustainability. The situation is further complicated by factors such as widespread over-the-counter sale of antibiotics and long turnaround times for test results, all of which hinder the effectiveness of AI-based CDSS. Without addressing these issues, scaling up digital AS interventions becomes challenging, and the tools risk becoming isolated demonstration project pieces rather than widely implemented solutions.

As alluded to, LMIC have limited data on antimicrobial consumption. WHO initiated the Global Antimicrobial Resistance and use Surveillance System in 2015. Ninety-eight countries, territories, and areas are enrolled, and antimicrobial use and AMR data are available based on income level, route of administration, antimicrobial classes, etc.^34^ However, only 80% of countries that are enrolled can provide AMR data.^34^ While many programs collect antimicrobial consumption, significant challenges are associated with standardized reporting. Although many countries legally restrict antibiotic dispensing without a prescription, weak regulatory enforcement and contextual realities undermine stewardship data and efforts.^35,36^ Successes such as the Vietnam Resistance Network (now the National AMR Surveillance Network) are noteworthy and are models for successful operational implementation of a surveillance network with provision of laptops, access to WHONET software, microbiology training, multiple workshops, and help desk access.^37,38^

### Recommendations

A critical gap in LMIC healthcare infrastructure is the absence of routine diagnostic testing, particularly microbiological surveillance. Without the ability to conduct routine diagnostic tests and obtain cultures, healthcare providers are forced to rely on empiric therapy. This practice often contributes to the overuse and misuse of antibiotics. Even when microbiological surveillance systems are available, they are often underfunded, under-maintained, and inconsistent. As a result, prescribers have limited or no access to local resistance data, making it difficult to tailor antibiotics to the specific needs of their patients. Given LMIC are disproportionately affected by MDROs, prioritizing methods to collect data on AMR are also vital for ML AI products to function properly.^39^ Simple digital tools, such as electronic prescribing systems, offline-accessible CDSS, and mobile-based data collection platforms that collect and transmit real-time surveillance data even in areas with limited internet access, can serve as foundational steps toward digital transformation in AS. The Digital Health Action Plan in the Eastern Mediterranean Region highlights that the rapidly increasing use of smart phones and growing interest from government poses an opportunity for the future.^31^ Interventions such as Africa CDC’s initiative to build and integrate technical capacity for AMR surveillance and the World Bank’s Regional Disease Surveillance Systems Enhancement program to strengthen national and regional AMR surveillance in Africa are notable efforts.^40,41^ Similarly, the Drivers of Resistance in Uganda and Malawi (DRUM) Consortium uses a One Health approach to collect demographic, geospatial, clinical, animal husbandry, water, sanitation, and hygiene data to investigate practices that may interrupt extended-spectrum beta-lactamase transmission.^42^ Holistic approaches such as these are well-suited to improve appropriate utilization of antimicrobials across their spectrum of use.

Additionally, investment in training healthcare professionals and building the capacity to maintain digital tools is critical for ensuring that these technologies are sustainable and scalable. Middle-income countries may consider use of tele-stewardship if the primary issue is personnel related; a Brazilian study highlighted tele-ASP efforts including infectious disease specialist review of orders via a secure web portal that was associated with a significant increase in appropriate antimicrobial prescriptions and decrease in carbapenem-resistant *Acinetobacter baumannii* isolation.^43^ Regional collaborations or partnerships with international organizations should be utilized to train stewardship personnel online such as those through the Global Health Network. Ideally, these programs would be region specific to address the unique needs and drug availability in each respective country. Further prioritization of free online resources such as surveillance and audit tools, treatment guidelines, policy tools, and open access journals will strengthen ASPs worldwide.^44^

Implementation of AI in AS faces multiple ethical, technical, and operational challenges (Table 2).^45^ The main ethical issues are the violation of patient data privacy, algorithmic bias, particularly prejudicial biases that may disproportionately impact marginalized populations common in LMIC, and the lack of transparency in “black box” models.^6^ The availability of data such as laboratory tests and imaging included in models trained using HIC data sets may hinder the applicability and utility of AI’s use in LMIC. Other operational considerations are the lack of institutional standardization and EHR platform integration difficulties that hamper implementation of AI systems. Real-world deployment of these systems is limited by operational challenges such as funding constraints, IT infrastructure deficits, and need for clinician education and acceptance.

**Table 2.** Challenges to implementing technology in antimicrobial stewardship in low- and middle-income countries (LMICs)^39^

Barrier category	Description	Impact
**Ethical challenges**	Patient data privacy concerns Algorithmic bias affecting marginalized groups Lack of transparency in “black-box” AI models	Risk of violating patient rights Risk of reducing trust and acceptance Risk of worsening health inequities
**Data quality & availability**	Variable data quality Incomplete AMR data, esp. in primary care such as lack of OTC antimicrobial data and RDT	Low accuracy/reliability of AI predictions Poor clinical decision-making
**Technical & integration challenges**	Lack of institutional standardization Difficulty integrating AI with EMRs or lack of EMR	Fragmented implementation Increased workload Workflow disruption
**Operational constraints**	Limited funding Weak IT infrastructure Insufficient clinician training	Deployment delays/failure Low adoption Difficulty identifying errors
**User acceptance & alert fatigue**	Excessive/irrelevant AI alerts Skepticism about AI tools	Reduced clinician trust Burnout Low usage
**Dynamic nature of AMR**	Rapid resistance evolution needing frequent AI updates	Outdated recommendations Lower effectiveness of interventions

Addressing these challenges will require a multidisciplinary approach involving data scientists, clinicians, informaticians, and policymakers. The Global Initiative on Artificial Intelligence for Health established by the WHO is taking the lead in facilitating global governance for AI in health and acknowledges their ability to bring an additional focus to LMIC.^46^ The initiative recently published strategic priorities to advance AI across the global community. These priorities include ethical and regulatory guidance and outlining implementation and operational processes to allow sustainable AI use globally. Although this is a global initiative, the Global Initiative on Artificial Intelligence for Health recognizes that the path to AI sustainability will need to be country specific.

## Conclusion

AS initiatives around the world have grown exponentially with the ongoing threat of AMR. The advances in technology to support ASPs have allowed for increased efficiency and higher-yield interventions to decrease overall patient morbidity and mortality. AI shows early promise and allows for further personalized medicine and precision. However, further regulation and studies are needed before widespread integration of AI into ASPs. Additionally, closing the technological gap between different resource settings is crucial to ensure gains made in addressing AMR are sustained. Investment in low-cost, scalable, and context-appropriate AI and digital solutions that can function within the limitations of resource-limited systems is essential. To ensure ethical and sustainable adoption of this technology, it is critical to validate algorithms across diverse populations, emphasize transparency in model development, and tailor implementation strategies to the resource and infrastructural realities of LMIC. Without these, the global fight against AMR risks being undermined.
